# Steinmann pin retractor-assisted reduction with circle plate fixation via sinus tarsi approach for intra-articular calcaneal fractures: a retrospective cohort study

**DOI:** 10.1186/s13018-019-1405-5

**Published:** 2019-11-14

**Authors:** Bin Zhao, Wenqian Zhao, Isaac Assan

**Affiliations:** 1 Department of Orthopedics, Shouguang Hospital of Traditional Chinese Medicine, 3353#, Shengcheng Street, Shouguang, 262700 Shandong China; 2 Department of Traditional Chinese Medicine, The People’ s Hospital of Shouguang, 1233#, Jiankang Street, Shouguang, 262700 Shandong China; 3 School of International Education, Weifang Medical University, 7166 Baotong West Street, Weicheng District, Weifang, 261053 Shandong China

**Keywords:** Steinmann pin retractor, Sinus tarsi approach, Calcaneus fracture

## Abstract

**Background:**

Sinus tarsi approach and mini-calc plate have been used for intra-articular calcaneal fractures. However, the sinus tarsi approach has limited exposure to the lateral wall, which makes it challenging to obtain an excellent anatomic reduction of the calcaneal body. What is more! To restore the width of the calcaneal body entirely and prevent the heel varus simultaneously with mini-calc plate was tough as well. Aimed to solve the aforementioned problems, our study focused on using the Steinmann pin retractor for reduction and the circle plate for fixation via the sinus tarsi approach.

**Methods:**

From March 2017 to January 2019, 15 patients with closed calcaneal fractures were treated with the method of Steinmann pin retractor-assisted reduction and circle plate fixation via the sinus tarsi approach. All these patients received a positive postoperative clinical and radiological evaluation.

**Results:**

A postoperative follow-up was done for each of the 15 patients, and the following scores and parameters were observed: value of visual analogue scale (VAS) was 1.44 ± 0.63, and The American Orthopaedic Foot and Ankle Score (AOFAS) Ankle-Hindfoot score was 84.31 ± 5.03 at the last follow-up. The Böhler angle (30.81 ± 3.56°), width (37.83 ± 4.87 mm), length (87.4 ± 3.33 mm), and height (86.23 ± 5.36 mm) of the calcaneus were improved significantly in comparison with preoperative values (− 0.94 ± 10.06°, 45.67 ± 5.68 mm, 82.72 ± 5.54 mm, 76.32 ± 7.98 mm), and these parameters were maintained excellently after 6–19 months’ follow-up.

**Conclusion:**

Our present study suggested that Steinmann pin retractor-assisted reduction with circle plate fixation via the sinus tarsi approach may serve as a safe and effective method for Sanders type II and type III calcaneus fractures. The Böhler angle, height, length, and body of the calcaneus were excellently restored postoperatively and maintained at last follow-up and rare postoperative complications.

**Trial registration:**

This study has been registered. The unique identifying number is research registry 5092.

## Introduction

Calcaneus fractures are common fracturing mainly caused by a high falling. Displaced intra-articular calcaneus fractures draw more attention in clinical treatment, for its potentiality of leading to post-traumatic osteoarthritis and resulting in claudication or disability to the patient. Since it is difficult to obtain an excellent anatomical reduction by nonoperative treatment [1] and uncertainly maintaining the Böhler angle by percutaneous fixation [2], the open reduction and stable internal fixation (ORIF) for displaced intra-articular calcaneus has been considered to be the gold standard protocol for intra-articular calcaneus fracture.

The extensile lateral L-shaped approach has been used for a notable advantage of the lateral wall expose. While about 30% of patients experience some incision complications including edge necrosis, dehiscence, hematoma, or deep infection [3]. Minimal incision, including the sinus tarsi approach, has been widely used for the advantage of lowering the risk of wound complications [4]. However, sinus tarsi approach has limited extensive exposure to the lateral wall, which makes it challenging to obtain an excellent anatomic reduction of the calcaneal body, even by the manual traction with a percutaneous Steinmann pin. Mini-calc plate satisfied the rigid triangular fixation, but the coverage of the lateral wall provided by Mini-calc plate was limited in comparison with the ordinary plate. Acquiring maximum lateral wall coverage was essential for the plate to restore the width of the calcaneal body entirely and prevent the heel varus effectively. Due to the reasons mentioned above, selecting a minimal approach with rare incision complication, and choosing a rigid fixation to meet the demands, including triangular fixation, maximum lateral wall coverage, and convenient insertion via minimal incision simultaneously, has been a real challenge in clinical research of orthopedics [5].

In our study, the sinus tarsi approach and the circle plate were chosen for ORIF; besides that, the Steinmann pin retractor device was used. With these efforts, we have aimed to restore the integrity of posterior facets, the height, length, and width of the calcaneal, and acquire rigid fixation which allows for early time functional exercise and, above all, rare incision complication. Subsequently, we assessed the measuring results, including Böhler angle, Gissanes angle, height, length, and width of the calcaneus in the radiological evaluation, and functional outcomes in clinical evaluation.

## Material and method

### Patients

We retrospectively review the clinical data of 15 patients with intra-articular calcaneal fractures who were managed surgically by performing Steinmann pin retractor-assisted reduction with circle plate fixation via sinus tarsi approach in the department of orthopedics of Shouguang Hospital of Traditional Chinese Medicine between 2017 and 2019. All the patients were surgically treated at the same hospital by the surgeon (Bin Zhao).

All the patients involved in this study were male. The average age was 45.67 ± 12.10 years (range, 27 to 79 years). All the patients were involved in labor work. The right side was involved in 6 patients and the left side in 10 patients (an indication of 1 patient being bilateral). The injury was sustained from a fall from a height in 14 patients and a vehicle accident in 1 patient. One patient was with a complicated scaphoid fracture of the affected foot side and 2 patients with complicated sural nerve injury of the affected side. All cases were closed fracture and classified with Sanders classification preoperative, including 5 cases of type IIA, 2 cases of type IIB, 1 case of type IIC, 3 cases of type IIIAB, 4 cases of type IIIAC, and 1 case of type IIIBC. Seven patients had more than 5 years of tobacco history and were instructed to stop smoking until complete wound healing.

All patients received lateral, axial X-ray radiography and horizontal, coronal computed tomography(CT) scan, and the three-dimensional reconstruction of the affected side were obtained before surgery. The lateral X-ray radiography of the unaffected side was obtained during the operation by C-arm fluoroscopy, which can be used as a reference for reduction. Surgery was performed after an average duration of 6.81 ± 4.00 days after the injury until the wrinkle test positive.

### Surgical procedure

**Fig. 1:**
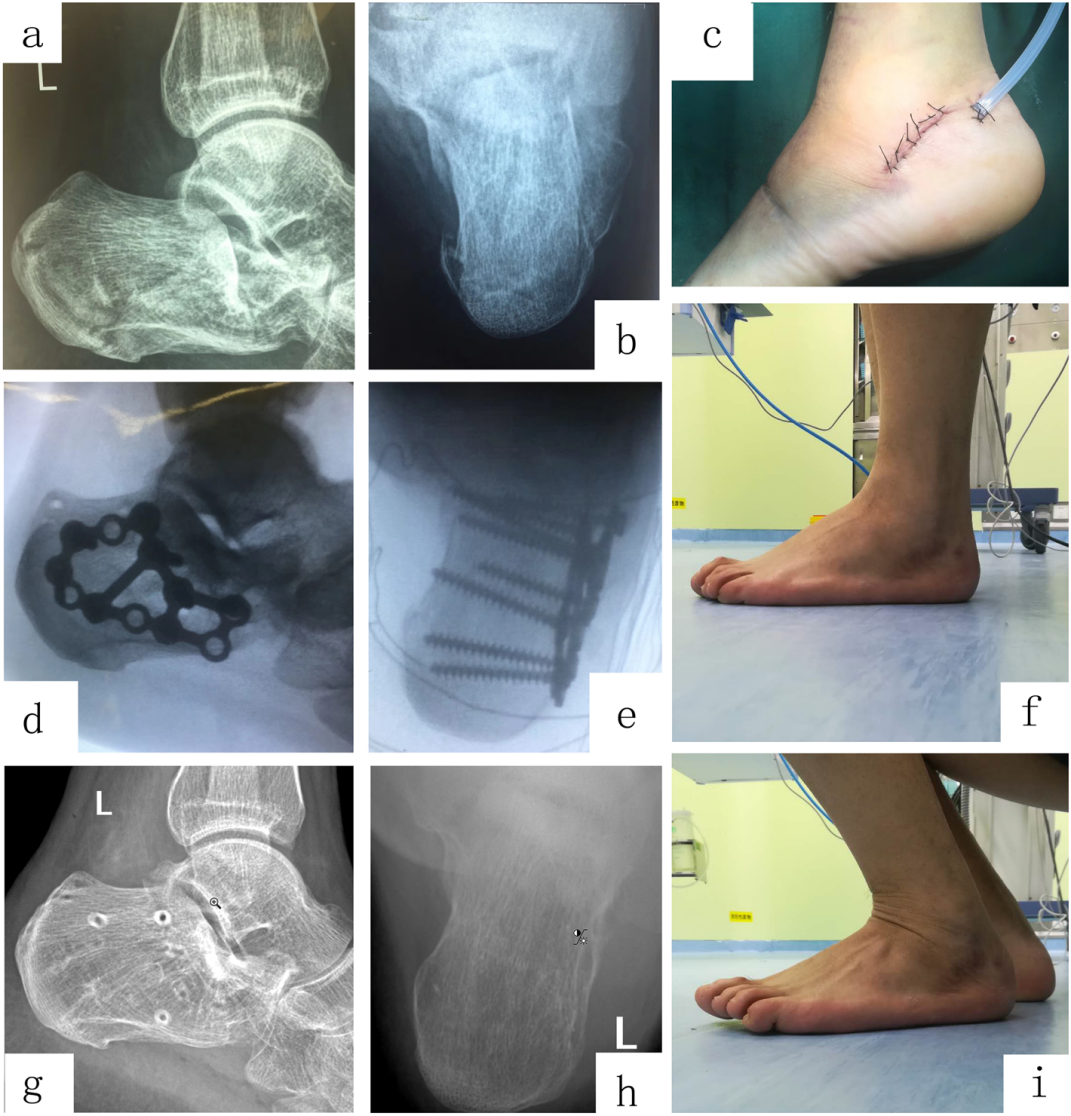
A 43-year-old male patient falls from a height of 3 m. A preoperative lateral radiograph (**a**) showing right calcaneus fracture with reduced Böhler angle (9°), length (81.9 mm), and height (70 mm), and the preoperative horizontal (**b**, **c**) computed tomography image showed Sanders type IIA fracture with increased calcaneal width (39 mm). The Steinmann pin retractor (**d**, **e**) was used during the operation. Postoperative lateral (**f**, **g**) and axial (**h**) radiographs show obvious correction of the Böhler angle (34°), and improvements in calcaneal length (88.8 mm), height (77.5 mm), and width (33 mm). AOFAS score was 89 at the last follow-up

**Fig. 2:**
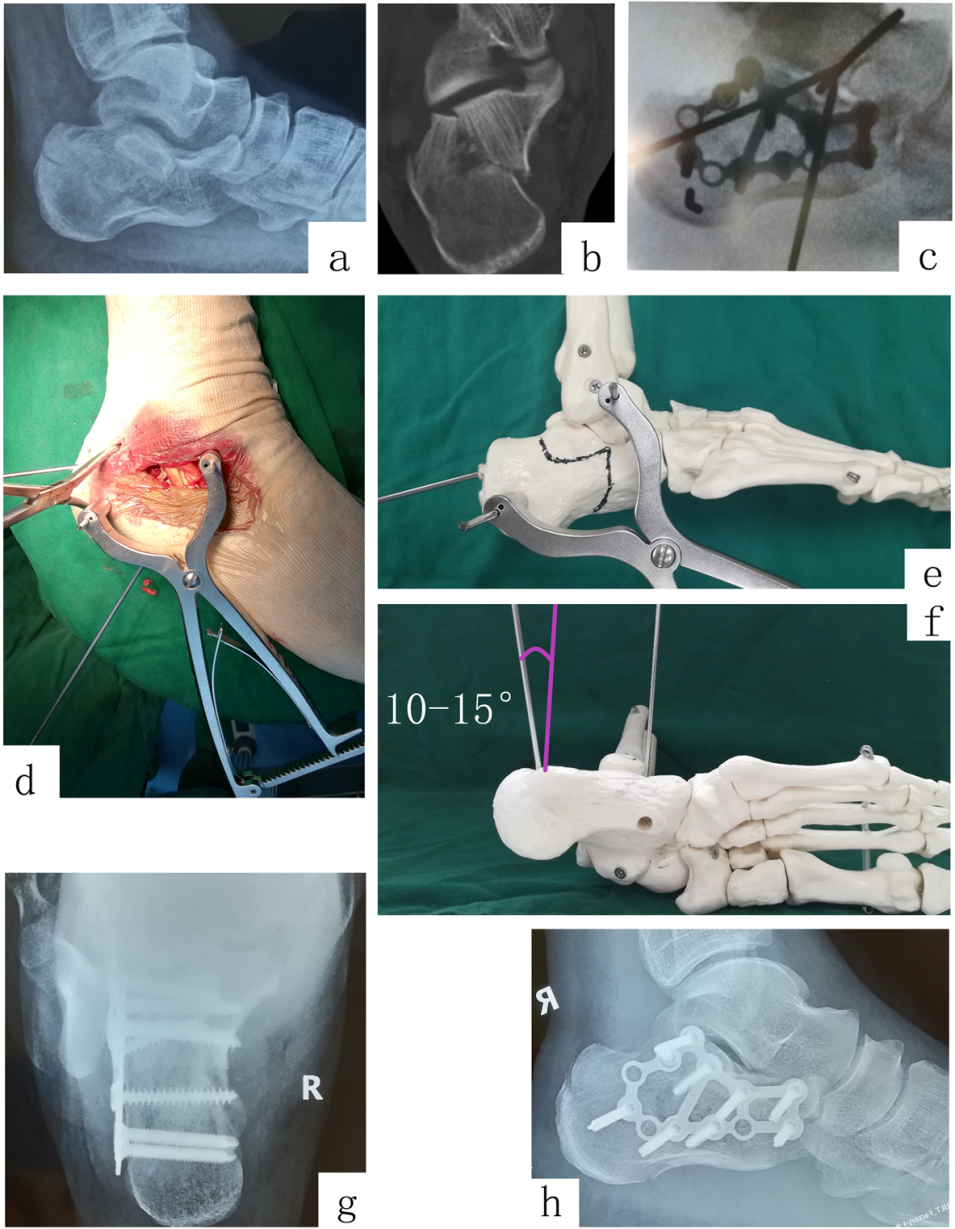
A 27-year-old male patient falls from a height of 2 m. Preoperative lateral (**a**) and axial (**b**) radiograph showed left calcaneus fracture with reduced Böhler angle (3°), length (80.1 mm), height (74.7 mm), and width (48.7 mm). The preoperative horizontal computed tomography image showed Sanders type IIB fracture. Steinmann pin retractor-assisted reduction with circle plate fixation via the sinus tarsi approach (**c**) was used. Postoperative lateral (**d**) and axial (**e**) radiographs show obvious correction of the Böhler angle (33°), and improvements in calcaneal length (84.7 mm), height (83.4 mm), and width (40.7 mm). The circle plate was removed (**g**, **f**) 17 months post-surgery, and AOFAS score was 87 at the last follow-up (**h**, **i**)

**Fig. 3:**
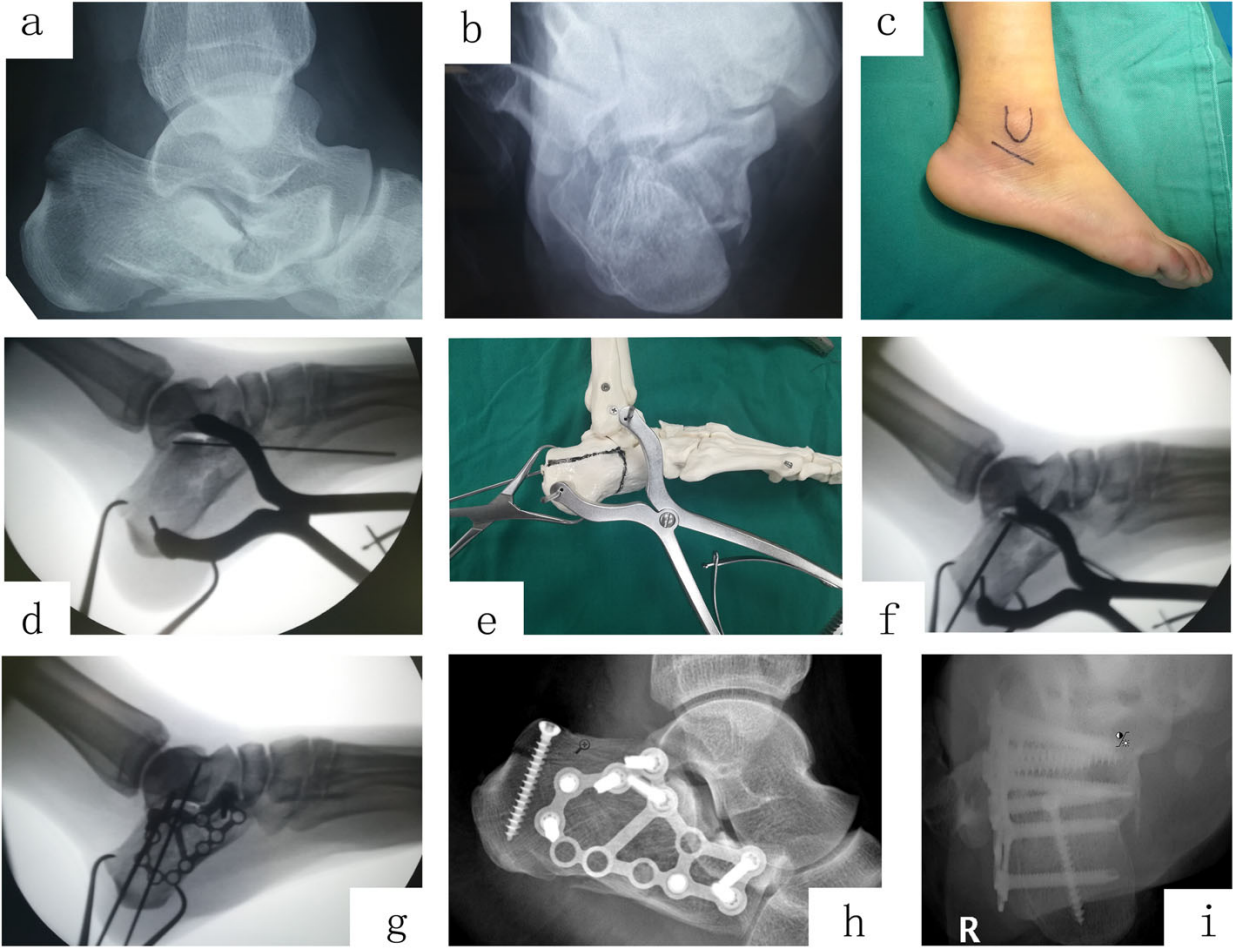
A 30-year-old male patient falls from a height of 1.5 m. Preoperative lateral (**a**) and axial (**b**) radiograph showed right calcaneus fracture with reduced Böhler angle (− 21°), length (75 mm), and height (61.1 mm), and width (59.3 mm). Steinmann pin retractor-assisted reduction with circle plate fixation via the sinus tarsi approach was used (**c**, **d**, **e**, **f**, **g**). Postoperative lateral (**h**) and axial (**i**) radiographs show obvious correction of the Böhler angle (32°), and improvements in calcaneal length (84.8 mm), height (80.5 mm), and width (40.4 mm). AOFAS score was 75 at the last follow-up

Surgery was performed under spinal-epidural anesthesia with the prone position. Firstly, preoperative preparations were made including the following: squeeze the body of the calcaneus with hands so as to restore the width of calcaneus partially and marked the sinus tarsi incision from the lower margin of the tip of the lateral malleolus to the proximal cuboid precisely on the skin, and prepare an electric pneumatic tourniquet (pressure, 40 KPa). Secondly, a 4–5-cm incision (Fig. 1c) was made accordingly after disinfecting by sharply dissect the sheath of the peroneus longus and brevis tendons from the lateral wall and protect the sural nerve carefully. Subsequently, two 3.0 Steinmann pins were drilled into talus and lateral process of calcaneal tuberosity, respectively. Gradually distract the Steinmann pin with the retractor (Huatrau, Chinatrau instrument Co`. Ltd., Guangzhou, China) (Fig. 2c, d) until the length and height of calcaneus were approximated to the unaffected side. However, it should be noticed that using a pointed reduction clamp to maintain the tubercular fracture blocks together before distraction is essential (Fig. 3d, e, f, g), especially in tongue-type fracture. Thirdly, the fatty tissue was removed from the tarsal sinus, to expose the posterior calcaneal facets. The retractor was over distracted until providing a 3–5-mm gap of joint space, which contributed to restoring the integrity of posterior calcaneal facets precisely. Subsequently, the pry-poking method was used to restore the Böhler and Gissanes angle according to the unaffected side and maintained these angels with Steinmann pin fixation temporary. Fourthly, moderate subperiosteal dissection along the lateral wall was performed until the circle plate (calcaneus plates 2, pure titanium, 12 holes, Suzhou Kangli Orthopaedics Instrument Co., Ltd., Suzhou, China) was inserted into the incision and achieved sound position under C-arm fluoroscopy. Cancellous bone screws were screwed-in from the front, top, to rear-like delta wing, which contribute to restoring the width of the calcaneus by the way, subsequently, screwed-in locking screws or cancellous bone screws via the incision or percutaneously for reinforcement. In the end, the Steinmann pins were removed, and the drainage tube was placed percutaneously before closing.

### Postoperative management and evaluation

Prophylactic antibiotics and non-steroidal anti-inflammatory drugs were administered to patients after surgery. The incisional area was pressurized with an elastic bandage 3–5 days after surgery, and the drainage tube was removed when the drainage liquid was less than 30 ml a day. The patients were encouraged to do toe flexion, and dorsiflexion actively post-anesthesia, and the “R.I.C.E” principle was carried out aimed to decrease the swelling postoperatively. Stitches were removed 2 weeks after surgery. Subsequently, full-day time exercises for the subtalar motion were encouraged, including ankle flexion and dorsiflexion exercises actively, and the circumduction, inversion, and eversion movements of the heel with hands passively. All of these efforts aimed to re-establish the motion of the subtalar joint. The affected side was allowed for partial weight-bearing with crutches 10 weeks after surgery until the evidence of the excellent condition of bridging fracture site reflected on the postoperative X-ray, and the absence of tapping pain along the axis of the calcaneal bone. Subsequently, full weight-bearing without crutches was carried out gradually over the next 6 weeks.

**Fig. 4:**
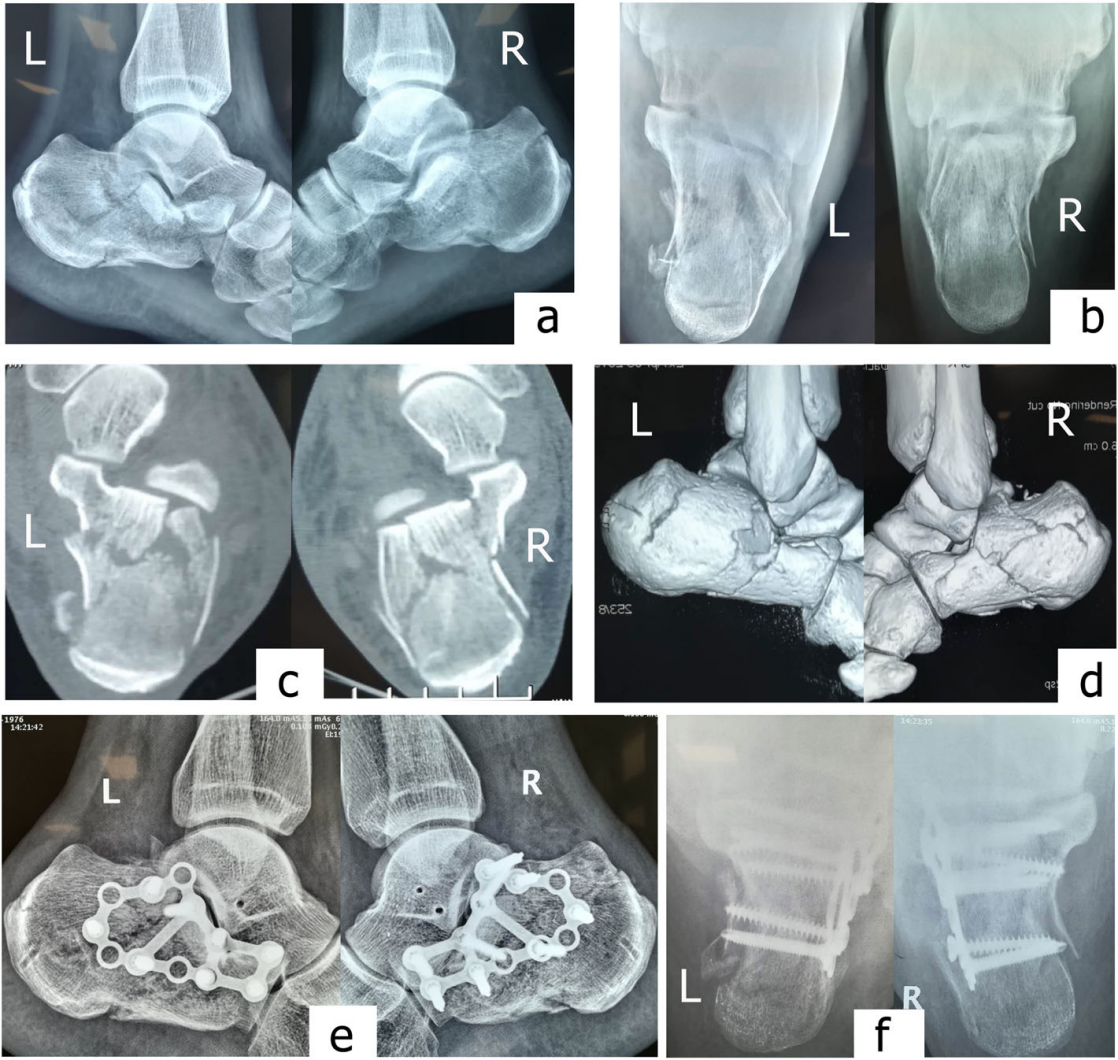
A 43-year-old male patient falls from a height of 4 m, over 10 years of smoking history. Preoperative lateral (**a**) and axial (**b**) radiograph showed bilateral calcaneus fracture with reduced Böhler angle (L: 2°, R: 5°), length (L: 80 mm, R: 75 mm), and height (L: 70 mm, R: 68 mm), and width (L: 44 mm, R: 42 mm). The preoperative horizontal computed tomography image (**c**) showed Sanders type IIIAC fracture. Steinmann pin retractor-assisted reduction with circle plate fixation via the sinus tarsi approach was used (**d**). Postoperative lateral (**e**) and axial (**f**) radiographs show obvious correction of the Böhler angle (L: 30°, R: 36°), and improvements in calcaneal length (L: 88.1 mm, R: 85 mm), height (L: 82.8 mm, R: 82.6 mm), and width (L: 36.2 mm, R: 36.9 mm). AOFAS score was 87 for left and 82 for right at the last follow-up

### Follow-up

Patients were evaluated clinically and radiologically at 1, 3, and 6 months postoperative. The clinical evaluation included a visual analogue scale (VAS) (0 = no pain, 10 = maximum imaginable pain) and the American Orthopaedic Foot and Ankle Society (AOFAS) Ankle-Hindfoot score. The radiological evaluation included axial and lateral X-ray, and the anatomical parameters of height, length, Böhler, and Gissanes angle were measured in the lateral view, while the calcaneus width was measured in the axial view. The anatomical parameters were measured with the PACS system (Picture Archiving and Communication Systems, version 2.5, Founder Group, Beijing, China). Clinical evaluation and radiological measurement were carried out by an independent physician who was not involved in the surgeries (Tables 1 and 2).

**Table 1:** Demographic data and clinical results of 15 patients

No.	Age (years)	Sanders classification	Side	Injury mechanism	Complication	The time from trauma to operation (days)	Operation duration (min)	Follow-up time (m)	AOFAS	vas
1	43	IIA	right	Falling from a height of 3 m		2	90	10	89	1
2	52	IIA	left	Falling from a height of 3 m		8	50	10	84	1
3	44	IIA	left	Falling from a height of 3 m		1	100	11	87	1
4	44	IIIBC	left	Falling from a height of 2 m	Left sural nerve injury	10	40	11	82	1
5	40	IIIAB	left	Falling from a height of 3 m		5	60	11	87	1
6	45	IIA	left	Traffic accidents	Left sural nerve injury, tarsal fracture	14	90	13	89	1
7	27	IIB	left	Falling from a height of 2 m		2	90	19	87	1
8	30	IIIAC	right	Falling from a height of 1.5 m		5	70	12	75	2
9	52	IIIAB	left	Falling from a height of 2 m		4	90	10	72	3
10	39	IIC	right	Falling from a height of 2 m		6	90	10	90	1
11	43	IIIAC	left	Falling from a height of 4 m		10	60	10	87	2
		IIIAC	right	Falling from a height of 4 m		10	60	8	82	2
12	79	IIA	right	Falling from a height of 1 m		14	60	8	82	2
13	58	IIIAB	right	Falling from a height of 2 m		4	90	6	82	2
14	42	IIA	right	Falling from a height of 2 m		6	90	6	87	1
15	47	IIIAC	left	Falling from a height of 2 m		8	90	6	87	1
mean ± SD						6.81 ± 4.00	76.25 ± 18.57	10.00 ± 3.16	84.31 ± 5.03	1.44 ± 0.63

Data are presented as a frequency count or mean ± SD

**Table 2:** Radiological results preoperative, postoperative, and the last follow-up

No.	Böhler angle preoperative (°)	Böhler angle postoperative (°)	Böhler angle at last follow-up (°)	Gissane angle preoperative (°)	Gissane angle postoperative (°)	Gissane angle at last follow-up (°)	Calcaneal body width preoperative (mm)	Calcaneal body width postoperative (mm)	Calcaneal body width at last follow-up (mm)	Calcaneal length preoperative (mm)	Calcaneal length postoperative (mm)	Calcaneal length at last follow-up (mm)	Calcaneal height preoperative (mm)	Calcaneal height postoperative (mm)	Calcaneal height at last follow-up (mm)
1	9	34	33	121	136	137	39	33	33.2	81.9	88.8	88.6	70	77.5	77.0
2	5	32	32	129	128	129	45	41.6	41.8	86.6	88	88.2	78.8	88.3	88.0
3	3	25	25	117	109	108	40.8	34.2	34.0	85.7	83.4	83.0	72.6	80.6	80.1
4	1	37	36	122	125	124	48.2	44.8	44.3	85.1	90.3	87.5	85	88.1	86.7
5	2	31	30	96	122	120	48.6	39.7	39.0	88.7	91.3	90.7	82.1	89.5	85.7
6	15	27	27	127	125	123	43.8	38.7	38.6	86.2	92	92.2	93.3	97.7	88.3
7	3	33	32	116	127	127	48.7	40.7	40.1	80.1	84.7	84.1	74.7	83.4	96.0
8	− 21	32	33	127	125	125	59.3	40.4	40.6	75	84.8	84.8	61.1	80.5	83.0
9	− 22	27	25	124	123	123	47	50	50.2	74.4	86	85.4	71.6	89.6	84.5
10	5	28	29	114	109	110	47.7	35.8	36.0	92.9	89	88.8	85	92.7	90.1
11	2 (left)	30	30	127	110	108	38.6	34.2	35.4	80	88.1	87.8	70	82.8	81.4
	5 (right)	36	35	120	110	112	36.6	34.9	35.2	75	85	85.4	68	82.6	80.2
12	− 4	27	27	122	116	118	45.7	37	37.4	82.1	85.7	84.8	77.1	87.4	86.6
13	− 13	30	30	104	106	108	51.9	35	36	76.8	80.1	80	71.7	80.9	80
14	− 2	29	29	129	125	125	41.4	32.2	32	84	89.7	89.9	78	91	90.4
15	− 3	35	34	103	112	113	48.3	33	33	89	91.5	91.5	82.1	87.1	87
mean ± SD	− 0.94 ± 10.06	30.81 ± 3.56*	30.43 ± 3.37*	118.63 ± 9.95	119.25 ± 8.91	119.38 ± 8.74	45.66 ± 5.68	37.83 ± 4.87*	37.93 ± 4.75*	82.72 ± 5.54	87.40 ± 3.33*	87.04 ± 3.32*	76.32 ± 7.98	86.23 ± 5.36*	85.31 ± 4.88*

Data are presented as a frequency count or mean ± SD. *P* < 0.05 is considered statistically significant. A comparison of data between groups was performed using a *t* test for paired data. *p < 0.05 vs. preoperative

### Statistical analysis

When applicable, all data were presented as means ± standard deviation. Comparison of data was performed using a *t* test for paired data. All statistical analyses were performed using the Statistic Package for Social Science(SPSS 19.0). Probability values < 0.05 were considered to be statistically significant.

## Results

All patients were followed-up. No case of sinus tarsi syndrome, compartment syndrome, or soft-tissue complications, such as wound edge necrosis, incision infection, hematoma, or new sural nerve injury in our study. All patients had a normal alignment of the calcaneus and stable plantigrade foot without signs of axial deviation or chronic swelling. Only two patients experienced moderate pain after walking of three blocks, but no patients need long-term use of painkillers. Three patients experienced subtalar joint stiffness without traumatic arthritis at the last follow-up. The symptoms of numbness in the innervation area was improved in two patients with preoperative sural injury (no obviously nerve rupture was observed during operation), and the quality of life was not disturbed by the numbness in these patients after surgery. The value of VAS was 1.44 ± 0.63, and the AOFAS Ankle-Hindfoot score was 84.31 ± 5.03 at the last follow-up. The radiological evidence judged the restoration of the joint surface with the incongruity of less than 2 mm, and with no screw loosening, implant break, nonunion, or malunion. The Böhler angle (30.81 ± 3.56°), width (37.83 ± 4.87 mm), length (87.4 ± 3.33 mm), and height (86.23 ± 5.36 mm) of calcaneus were significantly improved in comparison with preoperative (− 0.94 ± 10.06°, 45.67 ± 5.68 mm, 82.72 ± 5.54 mm, 76.32 ± 7.98 mm) (*p* = 0.000, *p* = 0.000, *p* = 0.000, *p* = 0.000 vs preoperative), and these parameters (30.44 ± 3.37°, 37.93 ± 4.75 mm, 87.04 ± 3.32 mm, 85.31 ± 4.88 mm) were well maintained after 6–19 months’ follow-up (*p* > 0.05 vs postoperative).

## Discussion

Sinus tarsi approach was initially described by Palmer [6] and was widely accepted for the treatment of intra-articular calcaneal fractures for the notable advantage of convenient exposure and the facilitating ability to restore the integrity of posterior facets. Above all, the sinus tarsi approach dramatically decreased the risk of wound complications in comparison with the laterally extended approach [4, 7]. However, poor visualization of the lateral wall via the sinus tarsi approach makes it difficult to restore the body of calcaneal anatomically. Supposed the length, height, and width of the calcaneus were not well improved, the late sequelae would occur inevitably; possibly anterior impingement associated with a significant loss of height and the lateral hindfoot pain associated with compression of the peroneal tendon sheath caused by severe shortening and widening of calcaneus [8]. In our study, Steinmann pin retractor provided a stable and accurate distraction during operation, by which we restored the length and height of the calcaneus under C-arm fluoroscopy according to the unaffected side; distracting the calcaneal tubercle persistently until the overlap between the fracture blocks disappeared under C-arm fluoroscopy or the outline of the calcaneus being restored was another object of reference for an excellent reduction, especially in bilateral calcaneal fractures (Fig. 4).

Steinmann pin retractor not only acts as a distractive device but also plays a significant role in preventing the heel varus. When the first Steinmann pin is driven into the talus, the second one is also driven into the body of calcaneus from the lateral process of tubercle and kept 10–15° varus to the previous one (Fig. 2f). After installing the retractor device, the second Steinmann pin is kept parallel to the previous one. Hence, the calcaneal body was present as varus status being corrected passively and valgus status maintained the process of distraction. However, the angle of the second Steinmann pin depends on the level of calcaneal varus.

After installing the retractor, providing a joint space view by the traditional methods, such as increasing the heel varus, was unsuitable. However, over-distracted appropriately with retractor contribute to providing a perfect surgical view for excellent reduction of the posterior facets. In our study, all the posterior facets were satisfactorily restored with the incongruity of less than 2 mm with the over-distracted method. However, we noticed that it was difficult to restore the sustentaculum tail fracture through the sinus tarsi approach, even with the assistance of the retractor. Hence, a previous study recommended for an additional medical approach to solve it [9]. Restricted by the prone position, no additional medial approach was performed in our study, for the patients with type IIC, IIIAC, and IIIBC fractures got a sound reduction in sustentaculum tail fracture, and this might be ascribed to the integrity of the medial soft tissue hinge (Additional file 1).

Circle plate fixation was used in extensile lateral L-shaped approach as usual but rare in the minimal incision, and the reasons why we chose this type of plate for fixation are as follows: Firstly, the circle plate provided maximum coverage of the lateral wall. Under the pressure of cancellous bone screws, the circle plate excellently restored the width of the calcaneal body. Secondly, the solid triangulate support by the front, top, and rear contribute to maintaining the height, length of the calcaneus, and the screws adjacent to the posterior facets benefited from maintaining the Böhler and Gissanes angle effectively. Thirdly, the circle plate can be inserted into the minimal incision easily. In our study, after 6–19 months’ follow-up, the Böhler angle, width, length, and height of calcaneus manifested no statistical difference in comparison with post-operation. However, every coin has two sides. Inserting a circle plate into the minimal incision indicated additional soft tissue detachment from the lateral wall in comparison with mini-calc plate, which potentially increases the risk of wound edge necrosis. Whilst there were no case of incision resulted in necrosis, it implied a satisfactory reason to ascribe to the careful subperiosteal dissection and the protection of the lateral branches of perforating peroneal artery attached to the lateral wall of calcanea [10, 11]. What is more, the bone healing process was not disturbed by subperiosteal dissection was confirmed by the previous study [12]. Indeed, the bone healing process manifested earlier than extraperiosteal dissection [12]. Sanders type IV calcaneus fractures were excluded in our study because neither sinus tarsi approach nor extended lateral approach achieved excellent outcomes in the previous research [13].

## Conclusion

Our present study suggests that Steinmann pin retractor-assisted reduction with circle plate fixation via sinus tarsi approach may serve as a safe and effective method for Sanders type II and type III calcaneus fractures. The Böhler angle, height, length, and body of the calcaneus were excellently restored postoperatively and maintained at last follow-up and rare postoperative complications.

## Additional file

**Additional file 1.** Ethical approval file, informed consent for patient and authorization for the use of the right of portrait.

## Data Availability

The datasets used and/or analyzed during the current study are available from the corresponding author on reasonable request.

## Abbreviations

AOFAS: The American Orthopedic Foot and Ankle Score
CT: Computed tomography
ORIF: Open reduction and stable internal fixation
PACS: Picture Archiving and Communication Systems
SPSS: Statistic Package for Social Science
VAS: Visual analogue scale
