# Supplementary material for: Steinmann pin retractor-assisted reduction with circle plate fixation via sinus tarsi approach for intra-articular calcaneal fractures: a retrospective cohort study
Source: J Orthop Surg Res. 2019 Nov 14;14:363. doi: 10.1186/s13018-019-1405-5 (PMC6854624; doi:10.1186/s13018-019-1405-5)
Supplement: Supplementary file 1 — Additional file 1. Ethical approval file, informed consent for patient and authorization for the use of the right of portrait. [file 13018_2019_1405_MOESM1_ESM.docx]

**Supplementary Information**

**1.** **Ethical approval**


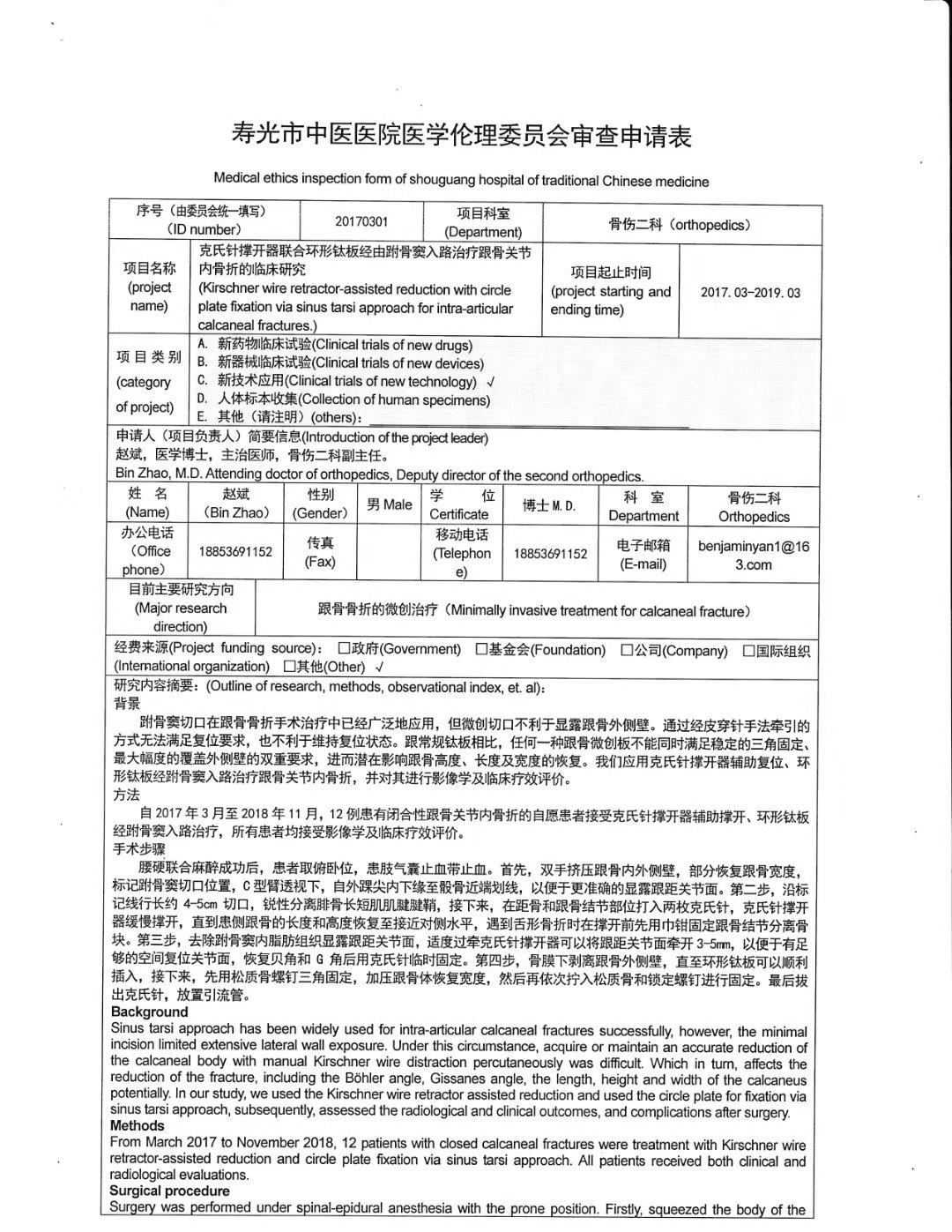


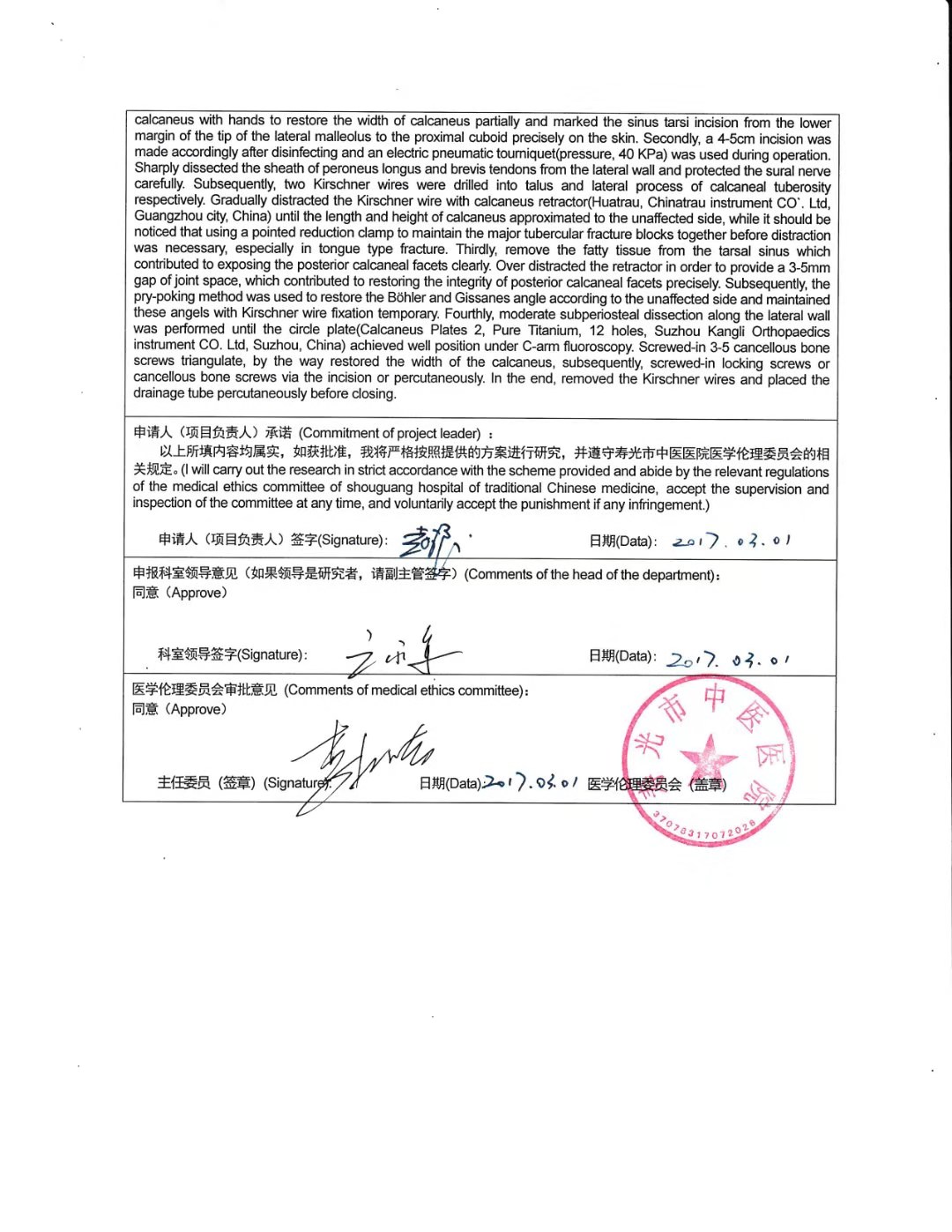


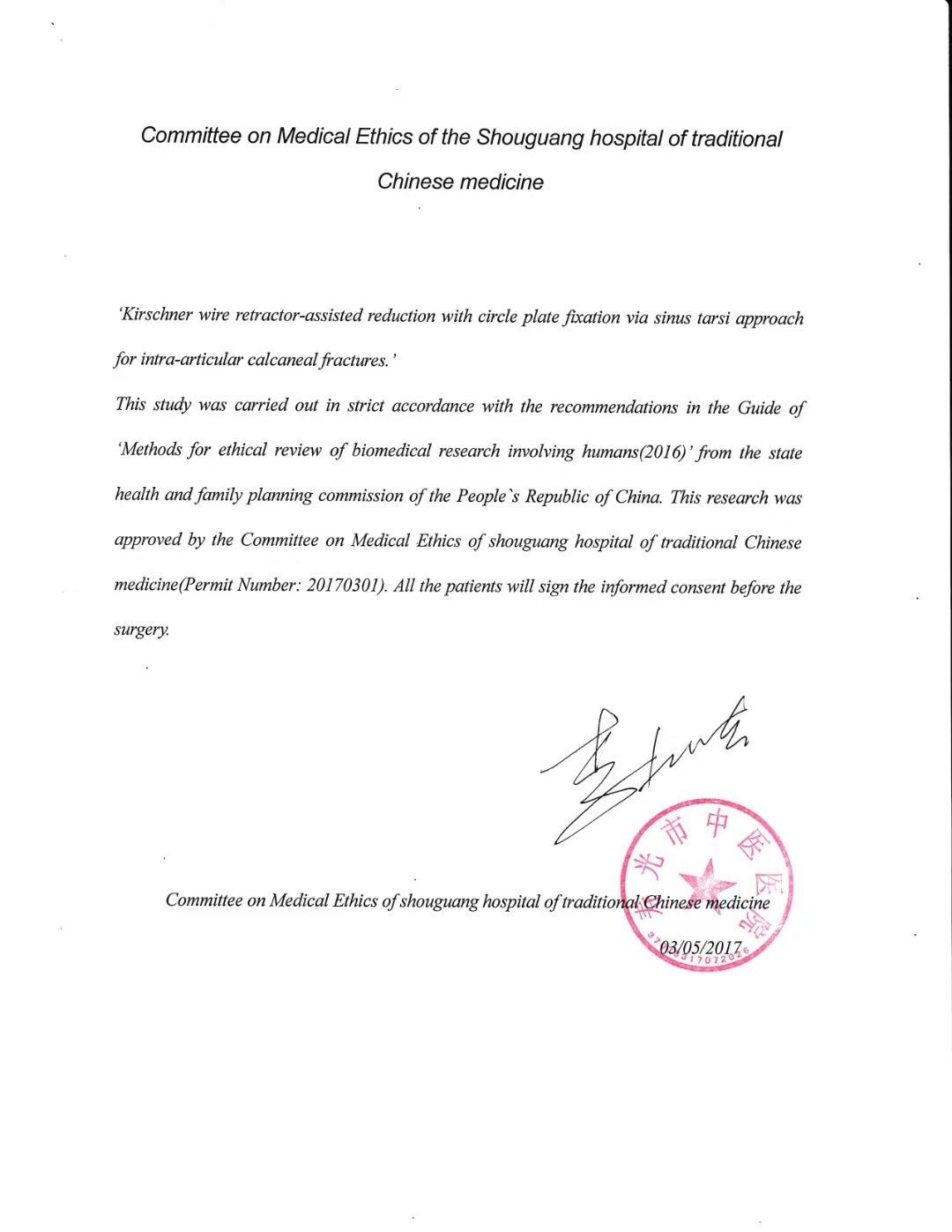


**2.** **informed consent for patient**

**
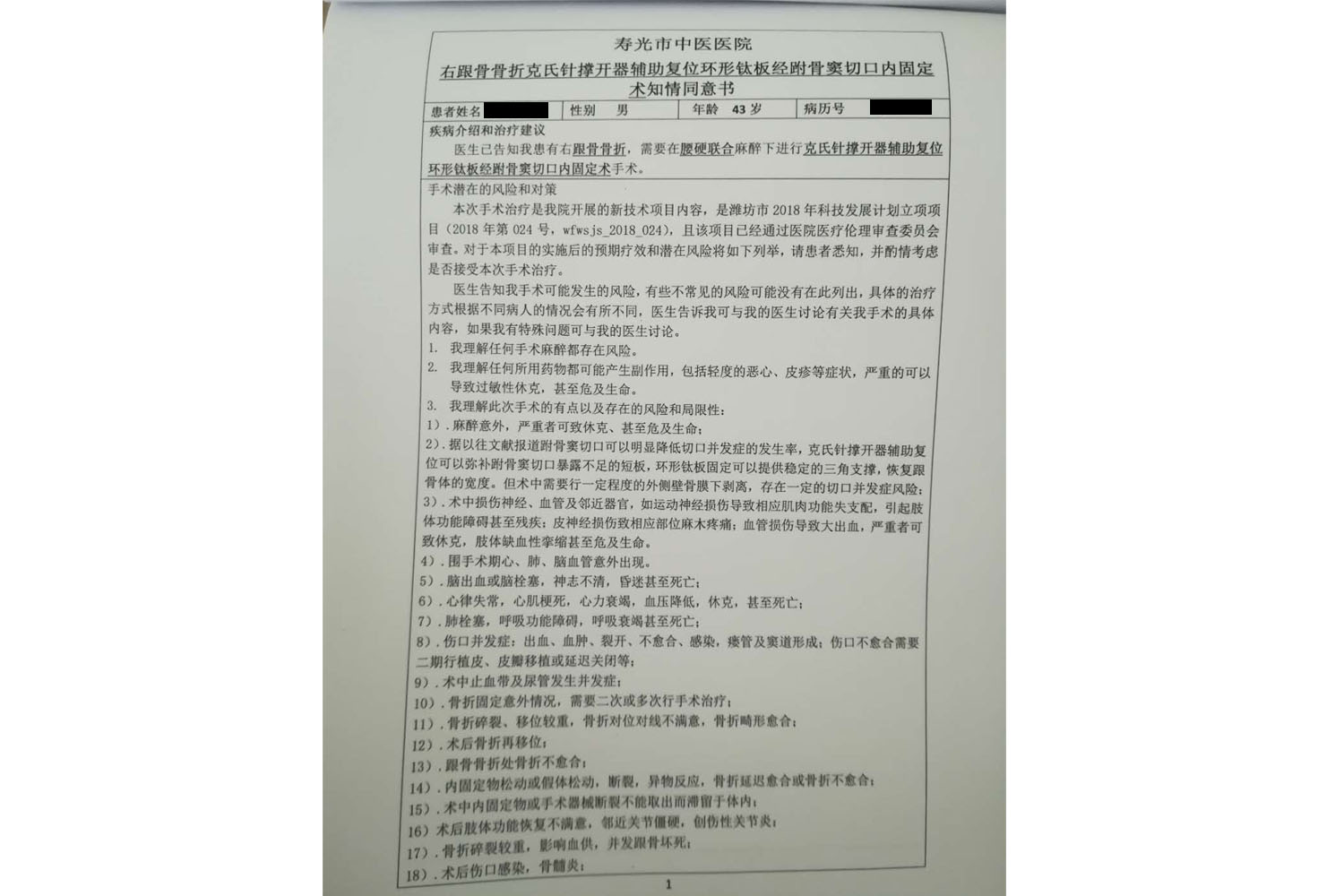
**

**
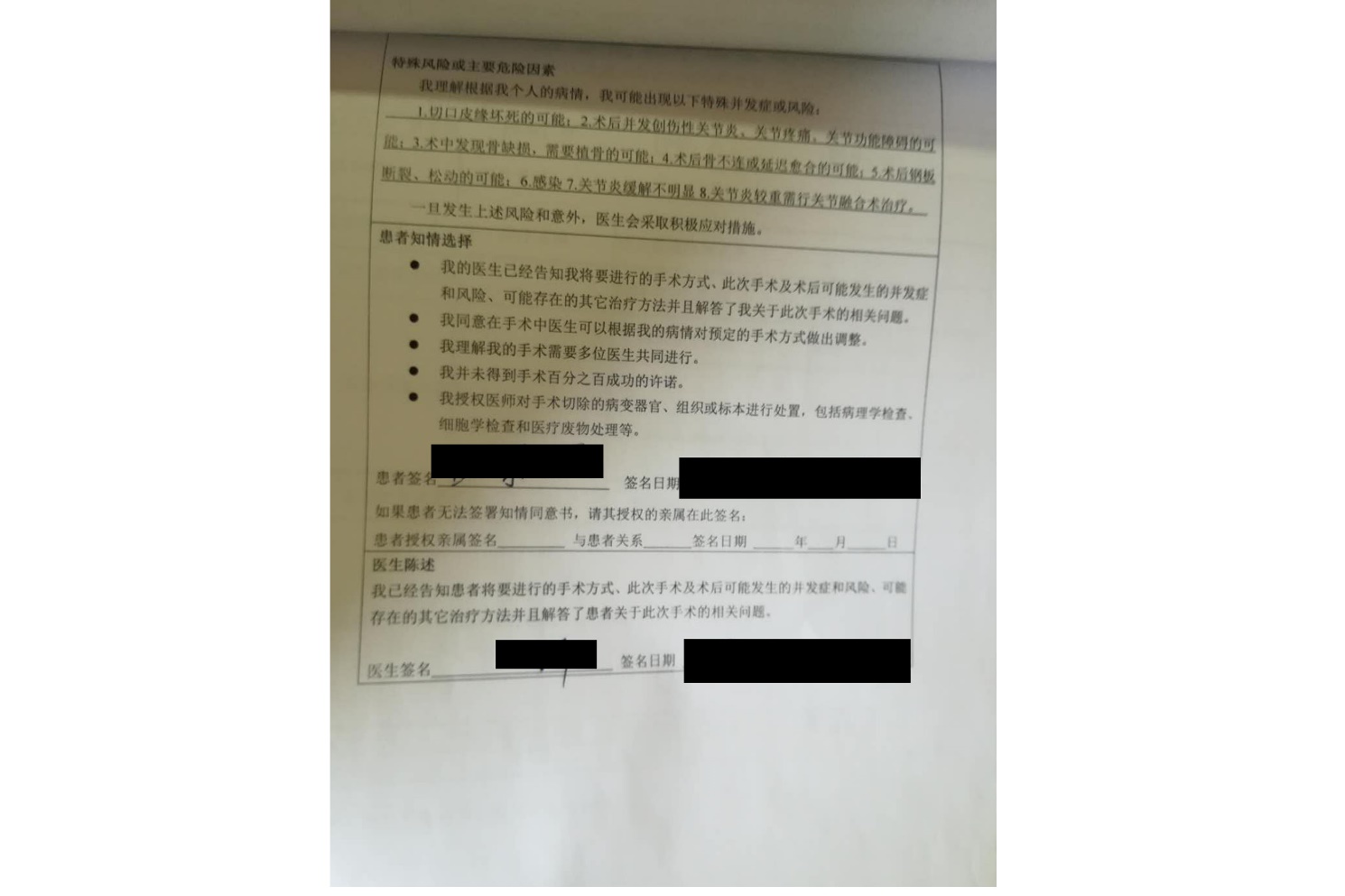
**

**3.** **Authorization for the use of the right of portrait**

**
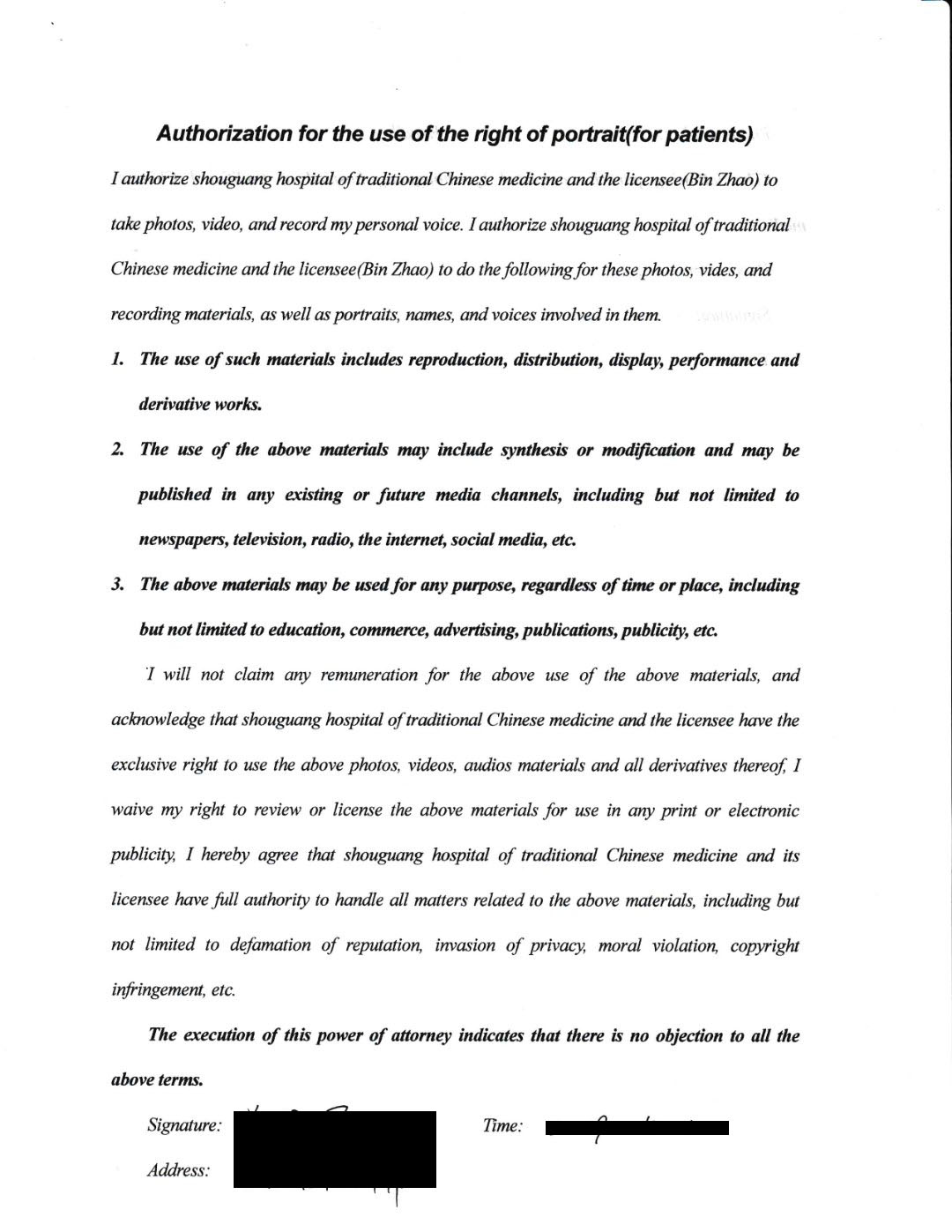
**

**
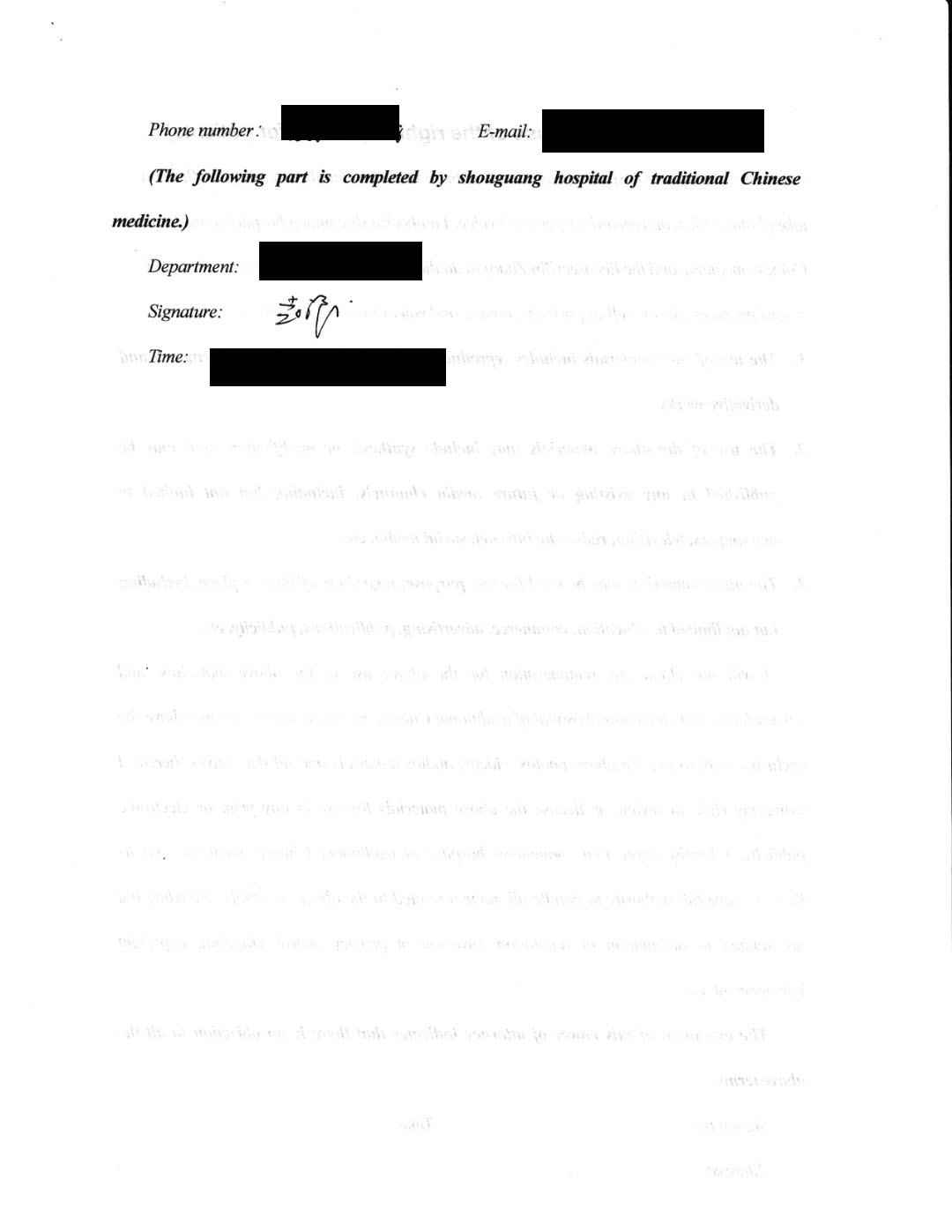
**

**
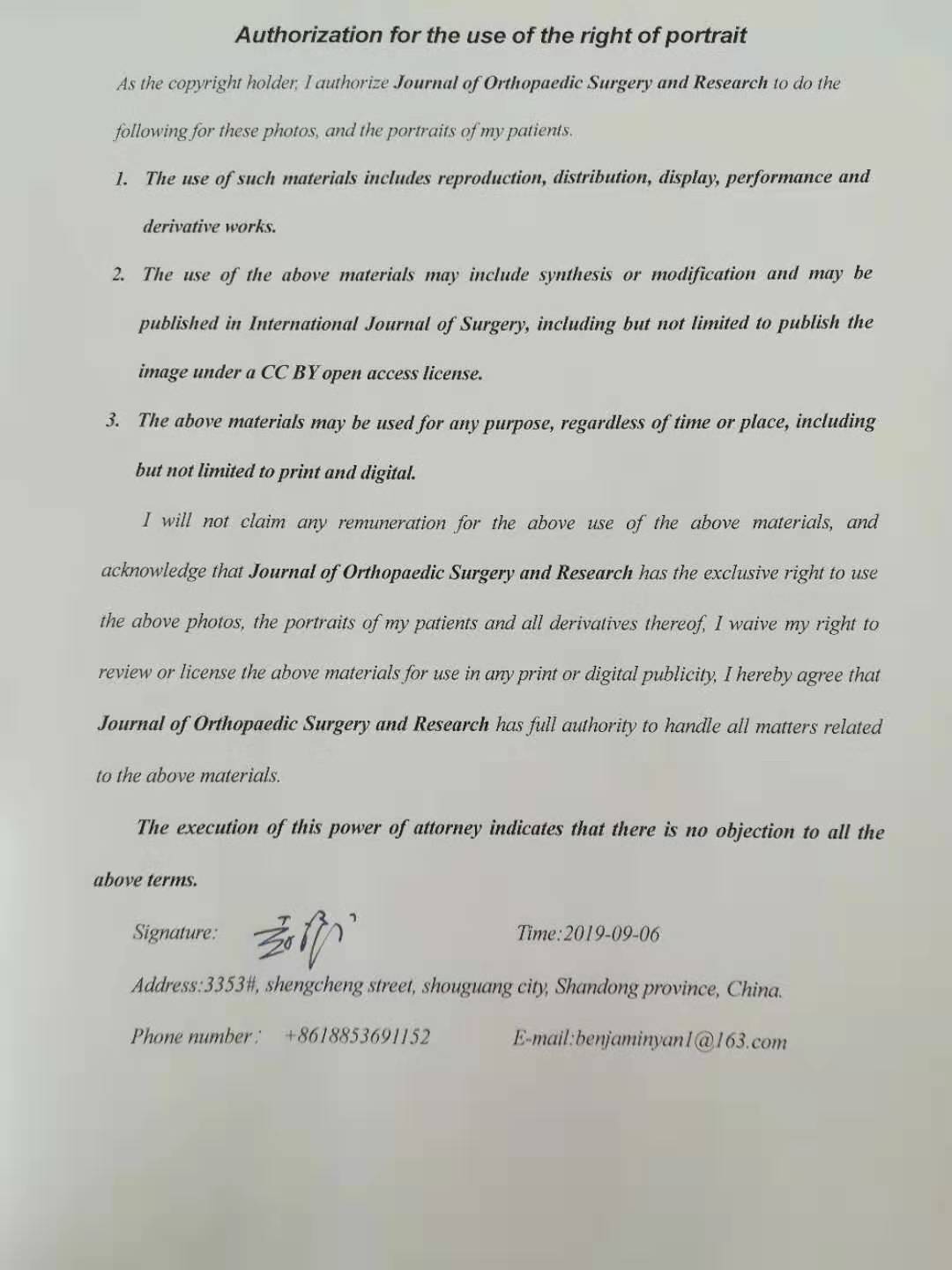
**
